# Cutaneous mastocytosis. Getting beneath the skin of the issue: a case report

**DOI:** 10.1186/1757-1626-2-69

**Published:** 2009-01-20

**Authors:** Parag M Tamhankar, Jyoti Suvarna, Chandrahas T Deshmukh

**Affiliations:** 1 Department of Medical Genetics, Sanjay Gandhi Postgraduate Institute of Medical Sciences, Lucknow, Uttar Pradesh, India; 2 Department of Pediatrics, Seth GSMC and KEM Hospital, Mumbai, Maharashtra, India

## Abstract

An eleven month old girl presented with chronic urticaria since three months of age. There was a generalised hyperpigmented maculo-papular rash. Darier sign was positive. The skin biopsy showed plenty of spindle shaped mast cells with eosinophilic cytoplasm infiltrating the dermis and the appendiceal structures. The diagnosis of cutaneous mastocytosis (urticaria pigmentosa) was made. The child received symptomatic relief with chronic oral hydroxyzine and ranitidine therapy. Automated epinephrine self-injectors usually prescribed in this condition for self-management of anaphylactic episodes were not available. Intramuscular administration of (1:1000) diluted adrenaline via a disposable tuberculin syringe was taught to the mother. A medical bracelet containing her diagnosis and instructions in emergency was custom-made for her.

## Background

Mastocytosis is a disorder characterized by clonal mast cell proliferation and accumulation within various organs, most commonly the skin. The cutaneous forms include generalized urticaria pigmentosa (UP)(commonest), solitary mastocytoma, diffuse erythrodermic form, telangiectatic macularis eruptive perstans (paucicellular) and pseudoxanthomatous form. Other types are: systemic mastocytosis (with/without skin involvement), mastocytosis in association with hematological disorders, lymphadenopathic mastocytosis with eosinophilia and mast cell leukemia[1,2].

## Case presentation

**Figure 1:**
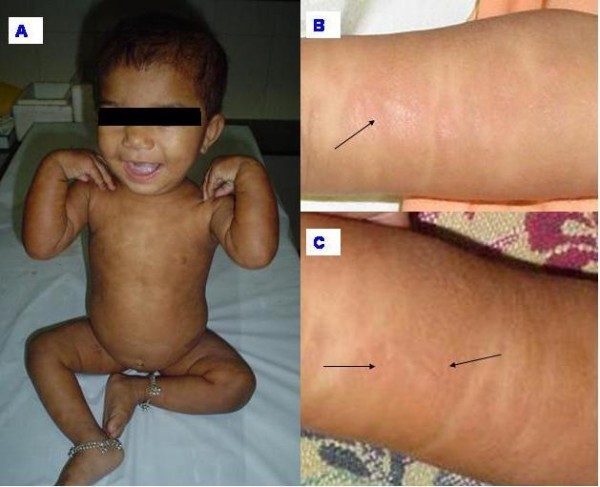
**(A) Generalised hyperpigmented maculopapular rash**. Palms, soles and face are also involved. (B) Darier sign: stroking the papular lesion linearly with a blunt instrument gives rise to linear wheal in (C).

**Figure 2:**
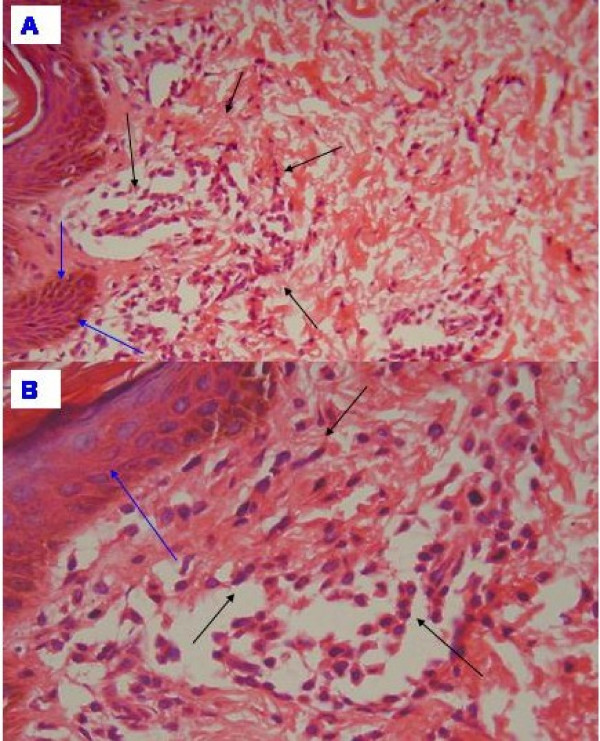
**Plenty of spindle shaped cells with eosinophilic cytoplasm i.e. mast cells infiltrate the dermis and the appendiceal structures (black arrows)**. The basal cells show more pigmentation (blue arrows) A:H & E, 10×; B: H & E, 40×.

An eleven months old girl presented with a generalized maculo-papular hyperpigmented pruritic rash since 3 months of age. The lesions became more prominent and itchy after a warm bath and in states of excitement (playing, crying etc.). Examination revealed multiple oval/round hyperpigmented papules and plaques distributed all over the body (face, trunk, extremities and acral areas too)(Fig. 1-A). Similar lesions were present on oral mucosa (cheeks and lips). Darier's sign (papular lesion becomes a palpable wheal after being vigorously rubbed) was positive.(Fig. 1-B &1-C) Pruritus was exaggerated after emotional excitement.** Patchy non-scarring scalp alopecia was present. There was no pallor, edema, lymphadenopathy or hepatosplenomegaly. The child's growth and development parameters were normal for her age. Skin biopsy showed plenty of spindle shaped mast cells with eosinophilic cytoplasm infiltrating the dermis and the appendiceal structures (black arrows). The basal cells showed more pigmentation (blue arrows).(Fig. 2) Complete hemogram, skeletal survey, liver function tests, blood coagulation profiles were normal. Bone marrow was normocellular.

The diagnosis was generalized cutaneous mastocytosis (Urticaria pigmentosa) (UP). Majority (75%) of UP present in the infantile age group [1,2]. Lesions may be present at birth. Initial lesions (brown/tan colored papules/nodules/plaque) involve the trunk and then spread centrifugally, symmetrically. Blistering/bullae formation may result from mast cell degranulation at dermo-epidermal junction. The other common cutaneous form: solitary mastocytoma is a large lesion (3–4 cm) presenting usually in infants; has no systemic effects and resolves fast. The rare diffuse erythrodermic form affects children below 3 years, causes diffuse skin thickening (peau-de-orange), may have serious systemic side effects and usually resolves completely (rarely systemic dissemination occurs). Telangiectasis macularis eruptiva perstans is rare form affecting adults, lesions are smaller, fewer and non-itchy. In systemic forms, organ infiltration by mast cells may lead to fracture, hepatosplenomegaly, lymphadenopathy, anemia, bleeding manifestations. [1,2]

Release of mast cell-derived mediators, such as histamine, prostaglandins, leukotrienes (D4, C4, E4), tumor necrosis factor, interleukins, platelet activating factors (common effects: vasodilatation, erythema, edema, pruritus, urticaria, bronchoconstriction, increased gastric acid, intestinal cramping, further degranulation of mast cells, leukocyte activation) may lead to headache, flushing, nasal stuffiness, dizziness, tachycardia, hypotension, syncope, anorexia, nausea, vomiting, abdominal pain, and diarrhea [3,4]. Classic signs such as Darier's sign and dermographism (ability to write (produce patterns) on skin by stroking macroscopically uninvolved skin) seen in our patient were due to release of above products locally [5]. The patchy scalp alopecia in our child could have occurred due to inflammation produced by mast cell infiltration in the hair follicles [6].

The cutaneous finding of diffuse infiltration of mast cells in the dermis in our patient is characteristic of UP. Toluidine blue or Giemsa stains highlight the mast cell granules. Increased melanin in basal cell layer and melanophages in the upper dermis leads to hyperpigmentation. However the diagnosis of UP involves excluding systemic involvement as similar skin findings may be present. Various tests such as complete hemogram (to look for anemia, thrombocytopenia, thrombocytosis, leucocytosis and eosinophilia), bone marrow exam (to look for mast cells leukemia), liver function tests, radiological skeletal survey (osteolytic lesions, osteoporosis or osteosclerosis) are needed to exclude systemic involvement. These tests were normal in our patient and ruled out infiltration of organs other than skin. Special tests such as serum tryptase levels, 24 hour urinary N-methylhistamine, N-methylimidazoleacetic acid and prostaglandin D2 metabolites excretion were not available to us but are rarely needed for diagnosis. They correlate with the severity and extent of cutaneous/systemic involvement. [1]

Treatment involves combining H1 antihistamines (for pruritus, flushing, urticaria) and H2 antihistamines (to reduce acidity, diarrhea and other gastro-intestinal (GI) symptoms) [4]. Oral mast cell-stabilisers (cromolyn) given daily mitigate onset and frequency of cutaneous and GI symptoms. Systemic corticosteroids are used in ascites, malabsorption, and severe skin disease (bullous). Psoralen plus UV-A therapy (PUVA), rarely required in children, is used for persistent UP in adults for cosmetic improvement [7]. We treated our patient with oral ranitidine 1 mg/kg/dose twice a day and oral hydroxyzine 2 mg/kg/day eight hourly. This was to be taken on a long term basis. The treatment led to subjective improvement of symptoms after about three months [8].

Anaphylactic episodes and shock can be precipitated in this disorder by intake of certain pharmacological agents like aspirin, codeine and over-exposure to physical stimuli like cold or sunlight. The management includes administration of fluids, adrenaline, antihistamines and other pressor agents at a medical facility may be required [9]. Automated epinephrine self-injectors (Epipen^R^) usually prescribed to initiate immediate management of anaphylactic episodes by the patient/his caretakers at home were not available [10]. A disposable plastic tuberculin syringe (with 26 gauge needle) loaded with 0.3 ml (diluted with normal saline 1: 1000) adrenaline was given to the mother and intramuscular administration in the anterolateral part of thigh anterolaterally was taught to her. She was told to promptly administer the injection in case any of the danger signs of anaphylaxis were noticed and then report to a nearby medical facility. The syringe was stored away from light in a sterile container, at room temperature for a maximum of a week. The family doctor replaced a fresh loaded syringe every week. A medical bracelet containing her diagnosis and instructions in emergency was custom-made for her.

Parents were told to avoid triggering factors causing release of mast cell mediators such as insect stings, certain drugs (aspirin, alcohol, codeine, quinine, amphotericin B etc), rapid changes in temperature and rubbing skin lesions. [2].

On follow-up over two years the lesions had partly resolved. Her growth and development parameters were satisfactory. She had three episodes of possible anaphylaxis which were averted successfully by intramuscular administration of epinephrine by her mother. She had been taken to her family physician every time and he too promptly treated her isotonic saline boluses and antihistaminics.

## Conclusion

Urticaria pigmentosa is a rare cause of chronic urticaria in children. Prolonged treatment with antihistaminics may be required for symptomatic relief. The condition is usually self-limiting in young children. The parents and the local doctor treating the child need to be educated about this condition. Motivated parents could be safely taught administration of epinephrine using tuberculin syringe in case automated self-injectors are unavailable.

## Patient's perspective (as told by her mother)

Our baby had these blackish lesions which became red and swollen since she was three months old. She became all red and sweaty after crying or after a bath. It was as if the village witch had cast a spell on her. Our family doctor had no clue to this condition. So we came to our district hospital where the doctors diagnosed her with mastocytosis. At first we were very apprehensive about giving her medicines for such a long time and thought that we would loose our child. Learning intramuscular epinephrine was very difficult (I am a fourth grader and my husband a fifth grader; he is a farmer). But the doctors have instilled confidence in me. I have used this injection on three occasions in the last two years. According to the doctors this has saved my child's life.

## Competing interests

The authors declare that they have no competing interests.

## Authors' contributions

PMT, JS, CTD identified, diagnosed and followed up the patient. PMT and JS were responsible for conception and design and acquisition of data. PMT wrote the first draft. JS and CTD have been involved in revising it critically for important intellectual content. PMT would be the guarantor of the paper. All authors read and approved the final manuscript.

## Consent

Written informed consent was obtained from the patient's parents for publication of this case report and accompanying images. A copy of the written consent is available for review by the Editor-in-Chief of this journal.

## References

[B1] Valent P Diagnostic evaluation and classification of mastocytosis Immunol Allergy Clin North Am 2006 26 3 515 34 10.1016/j.iac.2006.05.002 16931291

[B2] Alto WA Clarcq L Cutaneous and systemic manifestations of mastocytosis Am Fam Physician 1999 59 3047 3054 10392589

[B3] Longley J Duffy TP Kohn S The mast cell and mast cell disease J Am Acad Dermatol 1995 32 545 61 10.1016/0190-9622(95)90336-4 7896943

[B4] Miner PB Jr The role of the mast cell in clinical gastrointestinal disease with special reference to systemic mastocytosis J Invest Dermatol 1991 96 40S 44S 10.1111/1523-1747.ep12469015 2002261

[B5] Surjushe A Jindal S Gote P Saple D Darier sign Indian J Dermatol Venereol Leprol 2007 363 4 17921630 10.4103/0378-6323.35751

[B6] Xu X Solky B Elenitsas R Cotsarelis G Scarring alopecia associated with mastocytosis J Cutan Pathol 2003 30 9 561 5 10.1034/j.1600-0560.2003.00093.x 14507404

[B7] Rodríguez-Granados MT Carrascosa JM Gárate T Gómez-Díez S Guimaraens-Juantorena D Consensus document on bath-PUVA therapy. The Spanish Photobiology Group of the Spanish Academy of Dermatology and Venereology Actas Dermosifiliogr 2007 98 3 164 70 10.1016/S0001-7310(07)70040-6 17504700

[B8] Heide R Beishuizen A De Groot H Den Hollander JC Van Doormaal JJ De Monchy JG Pasmans SG Van Gysel D Oranje AP Dutch National Mastocytosis Work Group Mastocytosis in children: a protocol for management Pediatr Dermatol 2008 25 4 493 500 10.1111/j.1525-1470.2008.00738.x 18789103

[B9] Kamajian G Felix J Acute mastocytosis: a potential dermatologic emergency J Am Osteopath Assoc 1993 93 792 6 8365928

[B10] Mascarenhas S Aszkenasy OM EpiPen training provided to parents of children with food allergies Arch Dis Child 2009 94 1 76 10.1136/adc.2008.146274 19103793

